# WSES consensus conference: Guidelines for first-line management of intra-abdominal infections

**DOI:** 10.1186/1749-7922-6-2

**Published:** 2011-01-13

**Authors:** Massimo Sartelli, Pierluigi Viale, Kaoru Koike, Federico Pea, Fabio Tumietto, Harry van Goor, Gianluca Guercioni, Angelo Nespoli, Cristian Tranà, Fausto Catena, Luca Ansaloni, Ari Leppaniemi, Walter Biffl, Frederick A Moore, Renato Poggetti, Antonio Daniele Pinna, Ernest E Moore

**Affiliations:** 1 Department of Surgery, Macerata Hospital, Italy; 2 Clinic of Infectious Diseases, Department of Internal Medicine Geriatrics and Nephrologic Diseases, St Orsola-Malpighi University Hospital, Bologna, Italy; 3 Department of Primary Care & Emergency Medicine, Kyoto University Graduate School of Medicine, Japan; 4 Institute of Clinical Pharmacology and Toxicology, Azienda Ospedaliera Universitaria Santa Maria Misericordia, Department of Experimental and Clinical Pathology and Medicine, University of Udine, Italy; 5 Department of Surgery, Radboud University Nijmegen Medical Centre, The Netherlands; 6 Department of Surgery, "Mazzoni" Hospital, Ascoli Piceno Italy; 7 Department of Surgical Sciences San Gerardo Hospital, Monza, University of Milano-Bicocca, Italy; 8 Department of Surgery, University of Ancona, Italy; 9 Department of General and Transplant surgery, St Orsola-Malpighi University Hospital, Bologna, Italy; 10 Department of Surgery, "Ospedali Riuniti" Hospital, Bergamo, Italy; 11 Department of Surgery, Helsinki University Central Hospital, Finland; 12 Department of Surgery, Denver Health Medical Center, USA; 13 Department of Surgery, University of Texas Health Science Center, Houston, USA; 14 Emergency Surgery Department, University of São Paulo, Brazil

## Abstract

Intra-abdominal infections are still associated with high rate of morbidity and mortality.

A multidisciplinary approach to the management of patients with intra-abdominal infections may be an important factor in the quality of care. The presence of a team of health professionals from various disciplines, working in concert, may improve efficiency, outcome, and the cost of care.

A World Society of Emergency Surgery (WSES) Consensus Conference was held in Bologna on July 2010, during the 1^st^ congress of the WSES, involving surgeons, infectious disease specialists, pharmacologists, radiologists and intensivists with the goal of defining recommendations for the early management of intra-abdominal infections.

This document represents the executive summary of the final guidelines approved by the consensus conference.

## Introduction

A World Society of Emergency Surgery (WSES) Consensus Conference was held in Bologna on July 2010, during the 1^st^ congress of the WSES, involving surgeons, infectious disease specialists, pharmacologists, radiologists and intensivists with the goal of defining recommendations for the early management of intra-abdominal infections.

This document represents the executive summary of the final recommendations approved by the consensus conference.

The Surgical Infection Society and the Infectious Diseases Society of America have recently generated guidelines for the diagnosis and management of complicated intra-abdominal infections on 2010 [1]. IDSA guidelines represent an important reference for the management of intra-abdominal infections.

WSES guidelines represent a further contribution on this debated topic by specialists worldwide. The recommendations are formulated and graded according to the Grading of Recommendations Assessment, Development and Evaluation (GRADE) hierarchy of evidence [2,3] summarized in Table 1.

**Table 1:** Grading of recommendations from Guyatt and colleagues [2]

Grade of recommendation	Clarity of risk/benefit	Quality of supporting evidence	Implications
1A			
Strong recommendation, high-quality evidence	Benefits clearly outweigh risk and burdens, or vice versa	RCTs without important limitations or overwhelming evidence from observational studies	Strong recommendation, can apply to most patients in most circumstances without reservation
1B			
Strong recommendation, moderate-quality evidence	Benefits clearly outweigh risk and burdens, or vice versa	RCTs with important limitations (inconsistent results, methodological flaws, indirect or imprecise) or exceptionally strong evidence from observational studies	Strong recommendation, can apply to most patients in most circumstances without reservation
1C			
Strong recommendation, low-quality or very low-quality evidence	Benefits clearly outweigh risk and burdens, or vice versa	Observational studies or case series	Strong recommendation but may change when higher quality evidence becomes available
2A			
Weak recommendation, high-quality evidence	Benefits closely balanced with risks and burden	RCTs without important limitations or overwhelming evidence from observational studies	Weak recommendation, best action may differ depending on circumstances or patient or societal values
2B			
Weak recommendation, moderate-quality evidence	Benefits closely balanced with risks and burden	RCTs with important limitations (inconsistent results, methodological flaws, indirect or imprecise) or exceptionally strong evidence from observational studies	Weak recommendation, best action may differ depending on circumstances or patient or societal values
2C			
Weak recommendation, Low-quality or very low-quality evidence	Uncertainty in the estimates of benefits, risks, and burden; benefits, risk and burden may be closely balanced	Observational studies or case series	Very weak recommendation; other alternatives may be equally reasonable

## Principles of sepsis management

Severe sepsis and septic shock are the leading causes of multiple organ failure and mortality in noncoronary intensive care units (ICUs) [4,5].

Unfortunately, despite tremendous basic and clinical research efforts, mortality from septic shock remains unchanged at greater than 50%.

In an effort to improve sepsis-related mortality, several organizations have outlined evidence-based guidelines (EBGs) for the management of severe sepsis and septic shock [6].

Physicians have known about the existence of sepsis for centuries.

In 1992, the American College of Chest Physicians/Society of Critical Care Medicine (ACCP/SCCM) Consensus Committee developed definitions of patients with sepsis and its related disorders [7].

This Consensus represents the first attempt to create a universal language for diagnosing and treating sepsis.

Sepsis is defined as systemic inflammatory response syndrome (SIRS), resulting from infection.

Identifying patients with severe sepsis early and correcting the underlying microvascular dysfunction may improve patient outcomes. If not corrected, microvascular dysfunction can lead to global tissue hypoxia, direct tissue damage, and ultimately, organ failure.

### Systemic inflammatory response syndrome (SIRS)

SIRS is a reference for the complex findings that result from a systemic activation of the innate immune response, regardless of cause.

It includes the presence of more than one of the following manifestations:

• Temperature > 100.4°F or < 96.8°F (> 38°C or < 36°C)

• Heart rate > 90 beats/min

• Tachypnea, as manifested by a respiratory rate > 20 breaths/min or hyperventilation, as indicated by a PaCO_2 _< 32 mm Hg

• Alteration of white blood cell count > 12,000 cells/mm^3^, < 4,000 cells/mm^3^, or the presence of > 10% immature neutrophils.

### Sepsis

Sepsis is defined by the American College of Chest Physicians/Society of Critical Care Medicine (ACCP/SCCM) as SIRS resulting from infection.

### Severe sepsis

Severe sepsis is sepsis associated with at least one acute organ dysfunction, hypoperfusion, or hypotension.

### Septic shock

Septic shock occurs when sepsis-induced hypotension persists despite adequate fluid resuscitation.

### Multiple organ dysfunction syndrome (MODS)

MODS includes altered functions of two or more organs in an acutely ill patient.

### Pathophysiology

Abdominal sepsis occurs as result of intra-abdominal infection.

The pathophysiology of sepsis takes origin from the outer membrane components of both gram-negative organisms (lipopolysaccharide [LPS], lipid A, endotoxin) and gram-positive organisms (lipoteichoic acid, peptidoglycan). These outer membrane components are able to bind to the CD14 receptor on the surface of monocytes. By virtue of the recently described toll-like receptors, a signal is then transmitted to the cell, leading to the eventual production of the proinflammatory cytokines, including tumor necrosis factor (TNF), interleukin 1 (IL-1), IL-6, IL-8, and gamma interferon (IFN-), as well as other inflammatory mediators such as prostaglandins, leukotrienes, platelet activation factor, and nitrogen and oxygen intermediates. Most of these immunological mediators present multiple biologic effects, play a critical role in inflammation and immune responses, and have been recognized as key mediators in the pathogenesis of infectious diseases and, more particularly, the pathophysiologic alterations observed in endotoxic shock. As a result of the vicious cycle of inflammation, cardiovascular insufficiency and multiple organ failure occur and often lead to death [8-10].

### Haemodynamic support

Septic shock is primarily maldistributive shock. Its pathogenesis involves a complex interaction among pathologic vasodilation, myocardial dysfunction, and altered blood flow distribution due to the inflammatory response to infection. It evolves into a progressive pathophysiological deterioration that culminates in hypotension poorly responsive to adequate fluid resuscitation accompanied by hypoperfusion and organ dysfunction.

It is associated with three major pathophysiological effects: vasodilatation, maldistribution of blood flow, and myocardial depression.

In septic shock, the absolute intravascular volume may be normal; however, because of acute vasodilatation, relative hypovolemia occurs. Differently from other types of shock that are primarily caused by decreasing intravascular volume (hypovolemic) or decreasing cardiac output (cardiogenic), a characteristic of septic shock is the maldistribution of blood flow in the microcirculation. In septic shock also myocardial depression may occur. The relative hypovolemia, myocardial depression, and maldistribution result in decreased oxygen delivery (DO_2_) and subsequent tissue hypoxia.

Rivers and coll. [11] demonstrated that a strategy of early goal-directed therapy (EGDT) decreases the in-hospital mortality of patients who are taken to the emergency department in septic shock.

An organized approach to the haemodynamic support to sepsis includes use of fluid resuscitation, vasopressor therapy and inotropic therapy.

Patients with severe sepsis and septic shock may present ineffective perfusion. Poor tissues perfusion may cause a global tissue hypoxia, often associated to an elevated serum lactate level. A serum lactate value greater than 4 mmol/L (36 mg/dL) is correlated with poorer outcomes, even if hypotension is not yet present. Fluid resuscitation should be started as early as possible.

According to the Surviving Sepsis Campaign guidelines [6] during the first 6 hrs of resuscitation, the goals of initial resuscitation of sepsis-induced hypoperfusion should include all of the following as one part of a treatment protocol:

• Central venous pressure 8 to 12 mm Hg

• Mean arterial pressure (MAP) >65 mm Hg

• Urine output >0.5 mL/kg/hr

• Central venous (superior vena cava) or mixed venous oxygen saturation >70% or >65%, respectively

The early hypovolemic phase of sepsis must be always treated by providing appropriate high volume resuscitation.

The Surviving Sepsis Campaign guidelines [6] recommend that fluid challenge in patients with suspected hypovolemia be started with > = 1000 mL of crystalloids or 300-500 mL of colloids over 30 mins. More rapid administration and greater amounts of fluid may be needed in patients with sepsis-induced tissue hypoperfusion.

As the volume of distribution is less large for colloids than for crystalloids, resuscitation with colloids requires less fluid to achieve the same goals. A colloid equivalent is an acceptable alternative to crystalloid. Crystalloids are less expensive [6].

When an appropriate fluid challenge fails, to restore an adequate arterial pressure and organ perfusion, therapy with vasopressor agents should be started.

Vasopressor drugs maintain adequate blood pressure and preserve perfusion pressure for optimizing flow in various organs.

Both norepinephrine and dopamine are the first-line vasopressor agents to correct hypotension in septic shock. Both norepinephrine and dopamine can increase blood pressure in shock states, although norepinephrine seems to be more powerful. Dopamine may be useful in patients with compromised cardiac function and cardiac reserve [12], but norepinephrine is more effective than dopamine in reversing hypotension in patients with septic shock. Dopamine has also potentially detrimental effects on the release of pituitary hormones and especially prolactin, although the clinical relevance of these effects is still unclear and can have unintended effects such as tachyarrhythmias.

Dopamine has different effects based on the doses [13].

A dose of less than 5 μg/kg/min results in vasodilation of renal, mesenteric, and coronary districts. At a dose of 5-10 μg/kg/min, beta-1-adrenergic effects increase cardiac contractility and heart rate. At doses about 10 μg/kg/min, alpha-adrenergic effects lead to arterial vasoconstriction and increase blood pressure. Its major side effects are tachycardia and arrhythmogenesis.

The use of renal-dose dopamine in sepsis is a controversial issue. In the past, low-dose dopamine was routinely used because of the possible renal protective effects. Dopamine at a dose of 2-3 μg/kg/min was known to stimulate diuresis by increasing renal blood flow.

A multicentre, randomised, double-blind, placebo-controlled study about low-dose dopamine in patients with at least two criteria for the systemic inflammatory response syndrome and clinical evidence of early renal dysfunction (oliguria or increase in serum creatinine concentration), was published on 2000 [14]. Patients admitted were randomly assigned a continuous intravenous infusion of low-dose dopamine (2 μg/kg/min) or placebo administered through a central venous catheter. Administration of low-dose dopamine by continuous intravenous infusion to critically ill patients at risk of renal failure did not confer clinically significant protection from renal dysfunction.

A meta-analysis of literature from 1966 to 2000 for studies addressing the use of dopamine in the prevention and/or treatment of renal dysfunction was published on 2001 [15]. The Authors concluded that the use of low-dose dopamine for the treatment or prevention of acute renal failure was not justified on the basis of available evidence.

Norepinephrine is a potent alpha-adrenergic agonist with minimal beta-adrenergic agonist effects. Norepinephrine can successfully increase blood pressure in patients who are septic and remain hypotensive following fluid resuscitation. Norepinephrine is effective to treat hypotension in septic shock patients. In many studies norepinephrine administration at doses 0.01 to 0.3 μg/kg/min has been shown may be effective [16,17].

On 1993 Martin and coll. [18] published a randomized trial comparing norepinephrine vs dopamine. 32 volume-resuscitated septic patients were given either dopamine or norepinephrine to achieve and maintain normal hemodynamic and oxygen transport parameters for at least 6 h. Dopamine administration was successful in only 31% of patients, whereas norepinephrine administration was successful in 93%. Of the 11 patients who did not respond to dopamine, 10 responded when norepinephrine was added to therapy. Serum lactate levels were decreased as well, suggesting that norepinephrine therapy improved tissue oxygenation.

Recently a prospective trial by Patel and coll. compared dopamine to norepinephrine as the initial vasopressor in fluid resuscitated 252 adult patients with septic shock [19]. If the maximum dose of the initial vasopressor was unable to maintain the hemodynamic goal, then fixed dose vasopressin was added to each regimen. If additional vasopressor support was needed to achieve the hemodynamic goal, then phenylephrine was added. In this study dopamine and norepinephrine were equally effective as initial agents as judged by 28-day mortality rates. However, there were significantly more cardiac arrhythmias with dopamine treatment.

The Surviving Sepsis Campaign guidelines [6] state that there is no sufficient evidence to suggest which agent is better as initial vasopressor in the management of patients with septic shock.

Phenylephrine is a selective alpha-1 adrenergic receptor agonist primarily used in anesthesia to increase blood pressure. Although studies are limited [20], its rapid onset, short duration, and primary vascular effects make it an interesting agent in the management of hypotension associated with sepsis, but there are concerns about its potential to reduce cardiac output in these patients.

Epinephrine is a potent α-adrenergic and β-adrenergic agent that increases mean arterial pressure by increasing both cardiac index and peripheral vascular tone. The chief concern about the use of epinephrine in septic patients is the potential to decrease regional blood flow, particularly in the splanchnic circulation. On 2003 De Backer and coll. [21] published a trial to compare effects of dopamine, norepinephrine, and epinephrine on the splanchnic circulation in septic shock. In patients with severe septic shock, epinephrine administration increased global oxygen delivery and consumption. It caused lower absolute and fractional splanchnic blood flow and lower indocyanine green clearance, validating the adverse effects of therapy with epinephrine alone on the splanchnic circulation.

Epinephrine administration can increase blood pressure in patients who are unresponsive to first-line agents. It increases heart rate, and has the potential to induce tachyarrhythmias, ischemia, and hypoglycemia. Because of its effects on splanchnic circulation and its propensity to increase lactate concentrations, epinephrine has been considered a second-line agent [22].

A large (330 patients) randomized clinical trial published on 2007 by Annane and coll. [23] compared therapy with norepinephrine plus dobutamine (whenever needed) with epinephrine alone in septic shock. There was no evidence for a difference in efficacy and safety between epinephrine alone and norepinephrine plus dobutamine for the management of septic shock.

Vasopressin is a peptide hormone synthesized in the hypothalamus and is then transported and stored in the pituitary gland. Vasopressin mediates vasoconstriction via V1-receptor activation on vascular smooth muscle and mediates its antidiuretic effect via V2-receptor activation in the renal collecting duct system. In addition, vasopressin, at low plasma concentrations, mediates vasodilation in coronary, cerebral, and pulmonary arterial circulations.

Vasopressin infusion of 0.01 to 0.04 U/min in patients with septic shock increases plasma vasopressin levels to those observed in patients with hypotension from other causes, such as cardiogenic shock. Increased vasopressin levels are associated with a lesser need for other vasopressors. Urinary output may increase, and pulmonary vascular resistance may decrease. Infusions of > 0.04 U/min may lead to adverse, likely vasoconstriction-mediated events [24].

A large multicenter, randomized, double-blind trial comparing vasopressin versus norepinephrine infusion in patients with septic shock was published on 2008 [25]. A total of 778 patients underwent randomization (396 patients received vasopressin and 382 norepinephrine) and were included in the analysis. Low-dose vasopressin did not reduce mortality rates as compared with norepinephrine among patients with septic shock who were treated with catecholamine vasopressors.

According to the Surviving Sepsis Campaign guidelines [6] low doses of vasopressin (0.03 U/min) may be effective in raising blood pressure in patients refractory to other vasopressors and may have other potential physiologic benefits. Terlipressin has similar effects but is long lasting.

Dobutamine is frequently used in septic shock patients as an inotropic agent to increase cardiac output, stroke index, and oxygen delivery (Do_2_). However, the lack of benefit, and even possible harm, of dobutamine administration to increase Do_2 _to supranormal values in critically ill patients has raised questions regarding its use in the treatment of septic shock. Surviving Sepsis Campaign guidelines [6] recommend that a dobutamine infusion be administered in the presence of myocardial dysfunction as suggested by elevated cardiac filling pressures and low cardiac output.

Early intervention and implementation of evidence-based guidelines for the management of severe sepsis and septic shock improve outcomes in patients with sepsis. However, this is contingent on the early identification of sepsis. The early identification of sepsis is challenging because many of the signs and symptoms of sepsis are nonspecific. It is therefore necessary to identify those patients at highest risk for the development of sepsis and heighten our awareness for the development of sepsis in this population. In order to document the incidence of sepsis, assess its risk factors, and determine its impact on mortality in a general surgery population, the American College of Surgeons National Surgical Quality Improvement Project (NSQIP) dataset was analyzed [4]. The 2005-2006 NSQIP dataset contains prospectively collected clinical data and outcomes on 152.490 patients collected from 121 academic and community-based hospitals.

The analysis of the 2005-2006 NSQIP dataset identified 4 major risk factors for the development of sepsis or septic shock in general surgery patients: (1) age older than 60 years, (2) need for emergency surgery, (3) presence of any of the NSQIP comorbidities, and (4) male sex. These findings emphasized the need for early recognition through aggressive sepsis screening and rapid implementation of evidence-based interventions for sepsis and septic shock in general surgery patients with these risk factors.

Recently an analysis of 2005-2007 NSQIP dataset documented the incidence, mortality rate, and risk factors for sepsis and septic shock compared with pulmonary embolism and myocardial infarction in the general-surgery population [5]. Of 363.897 general-surgery patients, sepsis occurred in 8350 (2.3%), septic shock in 5977 (1.6%), pulmonary embolism in 1078 (0.3%), and myocardial infarction in 615 (0.2%). Thirty-day mortality rates for each of the groups were 5.4% for sepsis, 33.7% for septic shock, 9.1% for pulmonary embolism, and 32.0% for myocardial infarction. The septic-shock group had a greater percentage of patients older than 60 years. The need for emergency surgery resulted in more cases of sepsis and septic shock than did elective surgery. The presence of any comorbidity increased the risk of sepsis and septic shock 6-fold and increased the 30-day mortality rate 22-fold.

The incidences of sepsis and septic shock exceed those of pulmonary embolism and myocardial infarction. The risk factors for mortality included age older than 60 years, the need for emergency surgery, and the presence of any comorbidity. These findings confirmed the need for early recognition of patients at risk via aggressive screening and the rapid implementation of evidence-based guidelines.

## Principles of surgical management

Source control encompasses all measures undertaken to eliminate the source of infection and to control ongoing contamination.

As a general principle, every established source of infection should be controlled as soon as possible. The urgency of intervention is determined by the rapidity of the evolution of clinical symptoms.

Control of the septic source can be achieved either by surgical or non surgical means.

Non-surgical interventional procedures imply percutaneous drainages of abscesses.

Ultrasound and CT guided percutaneous drainage of abdominal and extraperitoneal abscesses in selected patients are safe and effective [26-33].

However surgery is the most important therapeutic measure to control intra-abdominal infections.

Generally, the choice of the procedure depends on the anatomical source of infection, on the degree of peritoneal inflammation, on the generalized septic response and on the patient's general conditions.

Surgical source control entails resection or suture of a diseased or perforated viscus (e.g. diverticular perforation, gastroduodenal perforation), removal of the infected organ (e.g. appendix, gall bladder), debridement of necrotic tissue or resection of ischemic bowel.

In cases of IAI complicated by septic shock, a single operation may not be sufficient to achieve source control, necessitating re-exploration. Three methods of local mechanical management following initial laparotomy for source control are currently debated: open-abdomen, planned re-laparotomy and on-demand re-laparotomy.

Following removal of infected tissue, attention should always shift to the restoration of anatomy and functionality of the gastrointestinal tract.

## Principles of antimicrobial management

Antimicrobial therapy plays an integral role in the management of intra-abdominal infections, especially in critical ill patients where empiric antibiotic therapy must be delivered as early as possible: in fact inadequate antimicrobial therapy is one of the variables most strongly associated to unfavorable outcome [6,34].

The initial antibiotic therapy for IAIs is always empiric because the patient is often critically ill and microbiological data (culture and susceptibility results) usually take at least 48 hours to become fully available.

The decision tree for the antimicrobial management of intra-abdominal infections depends mainly on three factors:

• Presumed pathogens involved and risk factors for major resistance patterns

• Clinical patient's severity

• Presumed/identified source of infection.

To predict the main pathogens involved and the related resistance patterns, infections are to be classed as community or hospital acquired.

During the past 2 decades the incidence of hospital-acquired infection caused by resistant microorganisms has significantly risen, probably in relationship with high level of antibiotic exposure and increasing rate of patients with one or more predisposing conditions such as recent exposure to antibiotics, high severity of illness, advanced age, co-morbidity, degree of organ dysfunction, low albumin level, poor nutritional status, immunodepression and presence of malignancy.

The major pathogens involved in community-acquired intra-abdominal infection are *Enterobacteriaceae*, *Streptococcus spp *and anaerobes (especially *B. fragilis*). Within the healthcare-associated infections, the spectrum of microorganism involved is broader, encompassing not only *Enterobacteriaceae, Streptococcus spp. *and anaerobes, but also *Enterococcus spp *and *Candida spp.*

The data regarding the role of *Candida spp *are actually conflicting: in a prospective multicenter epidemiological study conducted in 25 French centers, including more than 330 cases of peritonitis with positive microbiological cultures, two thirds of the health care-associated infections were associated to *Enterobacteriaceae *and one third to *Enterococcus spp*, while the isolation rate of *Candida spp *was less than 5% [35].

In contrast, in an observational study involving over 1182 patients with reliable microbiological data, the two genera of pathogens isolated from more than 25% of healthcare-associated infections and more commonly than from community-acquired infections were *Enterococcus spp *(29%) and *Candida spp *(33%) [36]. Apart from its epidemiological relevance, the clinical weight of *Candida spp *in peritonitis is high, since the isolation of the yeast from peritoneal fluid proved to be a variable independently associated to higher morbidity and mortality in a multiple-center, retrospective, case-control study conducted in critically ill patients admitted to 17 French ICUs [37].

More recently the same group confirmed the high mortality of candidal peritonitis (38%) in a prospective survey related on 93 patients admitted to ICU [38].

Enterococci are frequently responsible for hospital-acquired IAIs. During the past 2 decades the incidence of hospital-acquired enterococcal infection has significantly risen, probably in relationship with high level of antibiotic exposure and increasing number of patients with variable levels of immunosuppresion. In the aforementioned French survey, the prevalence of enterococcal isolation was significantly higher in the nosocomial cases of peritonitis and a significant increased incidence of fatal cases of peritonitis with positive cultures for enterococci was reported (20% versus 9% - p < 0.003) [35].

The threat of antimicrobial resistance has been identified as one of the major challenges in the management of intra-abdominal infections. The emergence of multidrug-resistant bacteria and the scanty pipeline of new antibiotics to fight them are, as of today, a concern especially for gram negative microorganisms, as highlighted in a recent report from the European Antimicrobial Resistance Surveillance System [39].

Hospital-acquired IAIs are commonly caused by more resistant bacteria, although the level of resistance is significant also in the community acquired infections.

The Study for Monitoring Antimicrobial Resistance Trends (SMART) program has been monitoring the activity of antibiotics against aerobic Gram-negative intra-abdominal infections. Hawser and coll. [40] reported susceptibility levels of key intra-abdominal pathogens in Europe for 2008, and showed that the options for effective empirical therapy of intra-abdominal infection have significantly reduced.

Coque and coll. highlighted the growing threat posed by increasing prevalence of extended-spectrum beta-lactamase (ESBL) producing Enterobacteriaceae all over Europe, even in countries traditionally showing low prevalence rates of resistance [41]. Increase of this resistance pattern has led to a progressive expansion of carbapenems use, because this class of antibiotics was traditionally considered the last resort for managing ESBL producers *Enterobacteriaceae*.

The inevitably increased carbapenem consumption has been associated to increasing carbapenemase production among *Enterobacteriaceae*. The recent rapid spread of serine carbapenemase in *Klebsiella pneumoniae *(KPC) is now an additional major threat for antimicrobial therapy in hospitals worldwide, and stresses the concept that the use of carbapenems must be mandatorily optimized in terms of indication and exposure [42].

Also *Acinetobacter spp *have worldwide shown similar alarming rates of increasing resistance to antibiotics. Today, Carbapenem-resistant A. baumannii-producing oxacillinases retaining susceptibility to only colistin and tigecycline is an ominous reality in hospitals worldwide and compounding this problem is the paucity of new antibiotics under development to address it [43].

In hospital acquired IAIs also *P. aeruginosa *plays an important - although less critical than in other settings - role. The high intrinsic antibiotic resistance of this pathogen, together with its extraordinary capacity for acquiring additional resistances through chromosomal mutations, should be always taken into consideration.

Among multidrug resistant Gram positive bacteria, Enterococci remain a challenge despite the availability of large number of antimicrobial agents theoretically active against this species. The clinical management of enterococcal infection remains challenging, mainly because no single agent could be anticipated to exert strong bactericidal activity against them.

### Clinical patient's severity

This choice of the antimicrobial regimen poses serious problems for the management of critically ill patients. In patients with severe sepsis or septic shock an early correct empirical antimicrobial therapy has a significant impact on the outcome, independently by the site of infection [44].

This data confirm the results of Riché and coll. who demonstrated, in a prospective observational study involving 180 consecutive patients with secondary generalized peritonitis, a significantly higher mortality rate in septic shock (35 versus 8% for patients without shock) [45].

Recent international guidelines for the management of severe sepsis and septic shock (Surviving Sepsis Campaign) [6] recommend intravenous antibiotics within the first hour after severe sepsis and septic shock are recognized, use of broad-spectrum agents with good penetration into the presumed site of infection, and reassessment of the antimicrobial regimen daily to optimize efficacy, prevent resistance, avoid toxicity and minimize costs [6]. Full adherence to these recommendations requires that clinicians, in order to optimize antibiotic therapy in critically ill patients, be aware that it is not sufficient to make the correct choice on the basis of the anti-biogram or of a correct epidemiological evaluation of risk factor for microorganisms and resistance patterns, but it is also mandatory to implement timely administration of the right dose on the right schedule, according to the pathophysiological and immunological status of the patient and to the pharmacokinetics properties of the chosen drugs [46].

This concept is correct not only from a clinical point of view; in fact sub-optimal plasma levels of antimicrobials and/or suboptimal exposure to antimicrobials in the infection site represent the best condition to favor the emergence of resistant strains, with a consequent higher probability of therapeutic failure and increased human and social costs.

For example, in critically ill patients, higher-than-standard loading doses of b-lactams, aminoglycosides or glycopeptides should be administered to ensure optimal exposure at the infection site independently of the patient's renal function [47-49].

For lipophilic antibiotics such as fluoroquinolones and tetracyclines, the 'dilution effect' in the extracellular fluids during severe sepsis may be mitigated by the rapid redistribution of the drug from the intracellular compartment to the interstitium. In contrast to what happens with hydrophilic antimicrobials, standard dosages of lipophilic antimicrobials may frequently ensure adequate loading even in patients with severe sepsis or septic shock [47].

Once appropriate initial loading is achieved, daily reassessment of the antimicrobial regimen is warranted, because the pathophysiological changes that may occur could significantly affect drug disposition in the critically ill patients.

Conversely, it is less evident that higher than standard dosages of renally excreted drugs may be needed for optimal exposure in patients with glomerular hyperfiltration [47].

Therefore, selecting higher dosages and/or alternative dosing regimens focused on maximizing the pharmacodynamics of antimicrobials might be worthwhile, with the intent being to increase clinical cure rates among critically ill patients.

Indeed, different approaches should be pursued according to the mechanism of antimicrobial activity exhibited by each antimicrobial. Two patterns of bactericidal activity have been identified: time-dependent activity (where the time that the plasma concentration persists above the MIC of the etiological agent is considered the major determinant for efficacy) and concentration-dependent activity (where the efficacy is mainly related to the plasma peak concentration in relation to the MIC of the microorganism). In addition, these agents show an associated concentration-dependent post-antibiotic effect, and bactericidal action continues for a period of time after the antibiotic level falls below the MIC [50].

Beta-lactams, glycopeptides, oxazolidinones, and azoles exhibit time-dependent activity: the shorter the drug elimination half-life, the more frequent the daily dose fractioning must be. For these drugs the employ of intravenous continuous infusion, which ensures the highest steady-state concentration under the same total daily dosage, may be the most effective way of maximizing pharmacodynamic exposure [51-54].

On the other hand, quinolones, daptomycin, tigecycline, aminoglycosides, polienes and echionocandins exhibit concentration-dependent activity; therefore the entire daily dose should be administered in a once daily way (or with the lowest possible number of daily administrations) with the intent of achieving the highest peak plasma level. The use of extended-interval aminoglycoside dosing strategies for the treatment of moderate-to-severe infections encountered in critically ill surgical patients [55,56].

## Classifications

Intra-abdominal infections (IAIs) include a lot of pathological conditions, ranging from uncomplicated appendicitis to faecal peritonitis.

From a clinical viewpoint IAIs are classified into uncomplicated and complicated [57].

In uncomplicated IAIs the infectious process only involves a single organ and does not proceed to the peritoneum.

In complicated IAIs, the infectious process proceeds beyond the organ, and causes either localized peritonitis (intra-abdominal abscess), or diffuse peritonitis.

Peritonitis is classified into primary, secondary or tertiary peritonitis [58].

Primary peritonitis is a diffused bacterial infection without loss of integrity of the gastrointestinal tract. It is rare. It mainly occurs in infancy and early childhood and in cirrhotic patients.

Secondary peritonitis, the most common form of peritonitis, is acute peritoneal infection resulting from loss of integrity of the gastrointestinal tract or from infected viscera. It is caused by perforation of the gastrointestinal tract (e.g. perforated duodenal ulcer), by direct invasion from infected intra-abdominal viscera (e.g. gangrenous appendicitis). Anastomotic dehiscences are common causes of peritonitis in the postoperative period.

Tertiary peritonitis is defined as peritonitis that persists after more than one failed source control procedure [59].

Intra-abdominal infections are also classified into community-acquired intra-abdominal infections (CA-IAIs) and healthcare-acquired intra-abdominal infections (HA-IAIs). CA-IAIs are acquired in community, whereas HA-IAIs develop in hospitalized patients or residents of long-term care facilities. They are characterized by increased mortality because of both underlying patient health status and increased likelihood of infection caused by multi drugs resistant organisms [59].

Moreover, in the classification of IAIs should be mandatory to introduce a grading of clinical severity, well represented by the sepsis definitions.

The updated sepsis definition is based on several clinical and bioumoral variables [60].

As is well demonstrated, there is an increased risk of death moving from the sepsis status to that of severe sepsis [61], and it appears reasonable to assume the latter as the breakpoint between a condition of clinical stability and a critically ill patient.

## Diagnosis

Accurate physical examination and laboratory studies are able to identify most patients with intra-abdominal sepsis undergoing immediate laparotomy (1 C).

In the patient with abdominal sepsis early detection and treatment is essential to minimize complications [7].

Complicated intra-abdominal infections diagnosis is mainly a clinical diagnosis.

Abdominal pain may be acute or insidious.

Hypotension and hypoperfusion signs such as lactic acidosis, oliguria, and acute alteration of mental status are indicative of evolution to severe sepsis [7].

Abdominal rigidity suggests peritonitis and the need for urgent laparotomy.

Plain films of the abdomen are often the first imaging studies obtained in patients presenting with intra-abdominal infections.

Upright films are useful for identifying free air under the diaphragm (most often on the right) as an indication of a perforated viscus.

In adult stable patients not undergoing immediate laparotomy, computerized tomography (CT) is the imaging modality of choice for intra-abdominal infections in adults (recommendation 2 B).

Especially in children, the radiation associated with CT, should be always be considered.

In unstable patients not undergoing immediate laparotomy who may not undergo studies requiring them to leave the ICU or emergency room, then ultrasound is the imaging modality of choice (recommendation 2 B).

When patients are stable, computerized tomography (CT) is the imaging modality of choice for most intra-abdominal processes [62,63].

Computed tomography (CT) of the abdomen and pelvis, when it is possible to perform, remains the diagnostic study of choice for intra-abdominal infections. CT should be performed with enteral and intravenous contrast [64].

Unstable Patients may not undergo studies that require trips away from the ICU or emergency department. In these patients intra-abdominal septic source may be detected by ultrasound (US) [65].

In experienced hands, the ultrasound can reliably diagnose most acute abdominal conditions in most patients.

Abdominal ultrasound has the advantage of being portable and may be helpful in the evaluation of right upper quadrant (eg, perihepatic abscess, cholecystitis, pancreatitis), right lower quadrant, and pelvic pathology (eg, appendicitis, tubo-ovarian abscess, Douglas abscess), but the examination is sometimes limited because of patient discomfort, abdominal distension, and bowel gas interference [66].

The value of both CT and US in the diagnostic work-up for intra-abdominal infections has been fully studied in relation to acute appendicitis. A meta-analysis by Doria and coll. evaluated the diagnostic performance of ultrasonography (US) and computed tomography (CT) for the diagnosis of appendicitis in pediatric and adult populations. This meta-analysis found that pooled sensitivity and specificity for diagnosis of appendicitis in children were 88% and 94%, respectively, for ultrasound studies and 94% and 95%, respectively, for CT studies. Pooled sensitivity and specificity for diagnosis in adults were 83% and 93%, respectively, for ultrasound studies and 94% and 94%, respectively, for CT studies.

From the diagnostic performance perspective, CT has a significantly higher sensitivity than US in studies of children and adults; from the safety perspective, however, the radiation associated with CT, especially in children, should be always considered [67].

## Treatment

Schematically intra-abdominal infections have been divided into three groups.

• Community acquired extrabiliary intra-abdominal infections

• Community acquired biliary intra-abdominal infections

• Hospital acquired intra-abdominal infections

## Extra-Biliary Community-Acquired Intra-Abdominal Infections

### Source control

#### Gastro-duodenal perforation

In the case of a perforated peptic ulcer, surgery is the treatment of choice. In selected cases (pts younger than 70 ys old, no shock, no peritonitis, lack of spillage of the water-soluble contrast medium at gastroduodenogram) non-operative management may be attempted. After initial non operative management, no improvement of conditions within 24 hours is indication to surgery (Recommendation 1 A).

In case of perforated peptic ulcer, surgery is considered the standard method of source control [68,69], also because postoperative mortality and morbidity rates have improved significantly [70].

Studies about the natural history of gastroduodenal ulcer perforation between the second half of 19^th ^and the first half of 20^th ^century [71,72] reported that perforations of the stomach were sealed by adhesions to the surrounding viscera preventing leakage from the stomach into the peritoneum.

In 1946, Taylor presented the first series of successful outcome of patients with perforated peptic ulcer conservatively treated [73]. Nowadays conservative treatment, also known as "Taylor method", consists of naso-gastric aspiration, antibiotics, intravenous fluids and *H. pylori *eradication therapy [74-76].

Patients older than 70 years old are significantly less like to respond to conservative treatment than younger patients [77]; also major medical illness, shock on admission and longstanding perforation (>24 hrs) are significantly associated with higher mortality rate in case of perforated peptic ulcer [78-80].

During non operative management, rapid deterioration or no improvement of clinical conditions within 24 hours from starting treatment are absolute indications to surgical treatment [81,82].

Finally, delaying the time point of operation beyond 12 h after the onset of clinical symptoms will worsen the outcome in perforated peptic ulcer [83].

Simple closure with or without omental patch is an effective and safe operation in case of small perforated ulcers (<2 cm). *H. pylori *status should be determined when the patients recover from the acute episode and the bacterium should be eradicated in those who are infected. (Recommendation 1 A).

In case of large perforated ulcers, concomitant severe bleeding or stricture, resective gastro-duodenal surgery may be required. The need for resection is established by surgeon based on intraoperative findings (Recommendation 1 B).

In case of small perforated gastroduodenal peptic ulcer, no significant differences in immediate postoperative course were reported after simple closure or definitive surgery [84-87].

Different suture techniques for simple closure of the perforation were described: simple closure by interrupted sutures [88] simple closure by interrupted sutures covered with pedicled omentoplasty, closure with a pedicled omental plug drawn into the perforation [89] and finally closure with a free omental patch [90].

Many patients in the published studies received omental patch repair rather than simple suture, but there was nearly no comparative evidence available to decide which repair technique is superior. A trial by Lau and coll. compared patch repair with fibrin sealing without finding any differences [91].

After closure alone, long term recurrence rate of peptic ulcer was significantly higher than after definitive surgery [92-95].

Eradication of Helicobacter pylori after simple closure and omental patch for perforated duodenal and gastric ulcers prevents recurrence.

To determine whether eradication of Helicobacter pylori could reduce the risk of ulcer recurrence after simple closure of perforated duodenal ulcer, a randomized controlled trial was conducted by Ng and coll. [96]. After 1 year, ulcer relapse was significantly less common in patients treated with anti-Helicobacter therapy than in those who received omeprazole alone (4.8% vs. 38.1%).

The first two cases of primary gastric resection for ulcer perforation were described by von Haberer as early in 1919 [97].

The method was used extensively for several decades but it is now rarely used for treatment of ulcer perforation. The role of resectional surgery in case of perforated peptic gastroduodenal disease is not well established; many reports advocate gastrectomy only in selected patients, in case of large gastric perforations, with concomitant severe bleeding or stricture [98-101].

Laparoscopic repair of perforated peptic ulcer is safe and effective in centers with experience (Recommendation 1 A).

The p.o. outcome of laparoscopic approach does not significantly differ from that of open surgery, except for lower analgesic p.o. request.

In all studies the patients had small ulcers (mean diameter 1 cm) and all patients received simple suture, mostly with omental patch, or sutureless repair.

No experience was reported with emergency laparoscopic resection or laparoscopic repair of large ulcers.

One systematic review [102], one meta-analysis [103] and three randomized controlled trials [104-106] comparing open and laparoscopic approach to gastroduodenal perforations were published. The postoperative course after laparoscopic repair did not significantly differ from that of open repair, except for lower analgesic request.

Biopsy and frozen section should be performed in all gastric perforations when a pathologist is available (Recommendation 2 C)

**If a patient has a curable tumor and acceptable general conditions (no shock, localized peritonitis, no comorbidities) the treatment of choice is gastrectomy (total or sub-total) with D2 lymph-node dissection; with poor general conditions and curable tumor is indicated a two-stage radical gastrectomy (first step simple repair and gastrectomy in a secondary elective intervention); with poor general conditions or non-curable tumor is indicated simple repair (Recommendation 2 C)**.

Treatment of choice of perforated gastric cancer is surgery. In most instances gastric carcinoma is not suspected as the cause of perforation prior to emergency laparotomy, and the diagnosis of malignancy is often made only by intraoperative or postoperative pathologic examination. The treatment should aim to manage both the emergency condition of peritonitis and the oncologic technical aspects of surgery.

Perforation alone does not significantly affect long term survival after gastrectomy [107], differed resection (i.e. two stage radical gastrectomy) does not affect long term outcome [108,109].

The presence of preoperative shock seems to be the most important negative prognostic factor for immediate postoperative survival after surgery for perforated gastric cancer [110].

Therefore, patients who have perforated gastric cancer should undergo appropriate gastric resection in spite of concurrent peritonitis unless the patient is hemodynamically unstable or has unresectable cancer [111-114].

#### Small bowel perforations

**In patients with small bowel perforations, surgery is the treatment of choice. (Recommendation 1 A)**.

**In case of small perforations, primary repair is preferable; when resection is required, the technique of anastomosis does not influence postoperative mortality or morbidity rates. (Recommendation 2 B)**.

**Laparoscopic approach should be performed by a laparoscopically experienced surgeon in selected institutions (Recommendation 2 C)**.

Primary repair of perforated bowel is preferable to resection and anastomosis because it carries a lower complication rate [115,116] even if the better outcome may reflect the limited tissue injury in these patients. Primary repair should not be performed in patients who have malignant lesions, necrotic bowel, perforations associated with mesenteric vascular injuries, or multiple contiguous perforations [117].

When resection is required, the entire diseased segment is resected, leaving healthy, well perfused ends for anastomosis. The technique for the enteroenterostomy, whether stapled or hand-sewn, seems to have little impact on the anastomotic complication rate [118,119].

Primary bowel anastomosis must be considered cautiously in the setting of gross purulent or feculent peritonitis because of a high rate of serious complications [120,121]. Laparoscopic management of small bowel perforations was reported [122] but there was no comparative study with open surgery.

#### Acute Appendicitis

Acute appendicitis is the most common intra-abdominal condition requiring emergency surgery.

The Surgical Infection Society and the Infectious Diseases Society of America have generated guidelines for the management and treatment of complicated intra-abdominal infections on 2010 [1].

**Operative intervention for acute, non-perforated appendicitis is the treatment of choice. Non-operative management of patients with acute, non-perforated appendicitis can be considered if there is a marked improvement in the patient's condition prior to operation (Recommendation 1 A)**.

Antibiotic treatment has been shown to be effective in treating selected patients with acute appendicitis. Three randomized controlled trials (RCTs) have compared the efficacy of antibiotic therapy alone with that of surgery for acute appendicitis [123-125]. A meta-analysis of these RCTs concluded that while antibiotics may be useful as primary treatment for selected patients, antibiotics are unlikely to replace appendectomy at present [126]. Selection bias and crossover to surgery in the RCTs suggest that appendectomy is still the gold standard therapy for acute appendicitis. A support for a less emergent approach comes from clinical trials analyzing time to perforation, which indicate this to be an unusual early event [127,128].

**Both open and laparoscopic approaches to appendectomy are appropriate (Recommendation 1 A)**.

A systematic review that included 45 randomized trials compared the diagnostic and therapeutic effects of laparoscopic and conventional open appendectomy in the treatment of suspected acute appendicitis [129]. The most consistent findings were an approximately 50% reduction in wound infections but a threefold increase in intra-abdominal abscesses in the laparoscopic appendectomy group. However, subsequently, two large studies have shown that patients undergoing a laparoscopic technique were more likely to be readmitted within 28 days of surgery [130] and that the risk for a complication was higher in the laparoscopic appendectomy group with uncomplicated appendicitis [131]. Taken together, open appendectomy may be preferred, although laparoscopic appendectomy is useful in selected subgroups of patients. Use of either approach should be decided by the surgeon's expertise. The laparoscopic approach is useful for obese patients, elderly patients and patients whose diagnosis is uncertain, especially women of childbearing age.

**Patients with perforated appendicitis should undergo urgent intervention (Recommendation 1 C)**.

**Patients with a periappendiceal abscess can be managed with percutaneous image-guided drainage. Appendectomy is generally deferred in such patients (Recommendation 1 A)**.

If imaging studies demonstrate a periappendiceal abscess, CT- or ultrasound-guided percutaneous drainage can often be performed. Percutaneous drainage with or without interval appendectomy to treat periappendiceal abscess results in fewer complications and shorter overall length of stay [132-134].

**The use of interval appendectomy after percutaneous abscess drainage or non-operative management of perforated appendicitis is controversial (Recommendation 2 C)**.

A survey using a postal questionnaire showed that 53% of surgeons performed routine interval appendectomy because they worried about recurrence [135]. However, the recurrence rate of appendicitis (10%-25%) and the complication rate of interval appendectomy (23%) are similar [135,136]. It was evident that the chances of missing malignancy are low and thorough investigation is better than interval appendectomy in detecting colonic cancer. These studies support the view that interval appendectomy is unnecessary in 75-90% cases.

#### Acute diverticulitis

Several major medical organizations, such as The American Society of Colon and Rectal Surgeons, The Society for Surgery of the Alimentary Tract, The American College of Gastroenterology, European Association of Endoscopic Surgeons, have proposed recommendations [137-141]. The practice parameters published by The American Society of Colon and Rectal Surgeons on 2006 are particularly useful [137]. The recommendations written here are generally consistent with them.

Complicated diverticulitis is defined as acute diverticulitis accompanied by abscess, fistula, obstruction, or free intra-abdominal perforation. Approximately 25% of patients diagnosed with diverticulitis for the first time present with complicated diverticulitis. Uncomplicated diverticulitis, accounting for 75% of cases, refers to diverticulitis without the complications noted above.

Hinchey Classification is used to describe perforations of the colon due to diverticulitis [142]. The classification is I-IV: Hinchey stage I - localized abscess (para-colonic), Hinchey stage II - pelvic abscess, Hinchey stage III - purulent peritonitis (the presence of pus in the abdominal cavity), and Hinchey stage IV - fecal peritonitis.

**Non-operative treatment, with bowel rest and antibiotics, is suggested in patients with uncomplicated diverticulitis (Recommendation 1 C)**.

Conservative treatment of acute uncomplicated diverticulitis is successful in 70 to 100 percent of patients [137]. Uncomplicated diverticulitis may be managed as an outpatient (dietary modification and oral antibiotics) for those without appreciable fever, excessive vomiting, or marked peritonitis, as long as there is the opportunity for follow-up. The patient should be able to take liquids and antibiotics by mouth. Hospitalization is indicated if the patient is unable to take liquids or has severe pain, or if symptoms fail to improve despite adequate outpatient therapy. Antibiotics should be selected to treat the most common bacteria found in the colon: gram-negative rods and anaerobic bacteria [143]. Non-operative treatment will resolve acute diverticulitis in 85 percent of patients, but approximately one-third will have a recurrent attack often within one year [144].

**The decision to recommend elective sigmoid colectomy after recovery from acute diverticulitis should be made on a case-by-case basis (Recommendation 1 C)**.

The overall rate of recurrence appears to be approximately 10 to 30% within a decade after a first documented attack and that the majority of patients who have a single episode of diverticulitis will not have another [145]. In one report involving an average follow-up of 9 years with 2551 patients whose initial episode of diverticulitis was treated successfully without surgery, only 13% had recurrent attacks and only 7% required colectomy [146]. These observations imply that routine elective colectomy is probably unwarranted if the disease is successfully managed on initial presentation and that surgical treatment should be limited to patients whose symptoms persist despite conservative therapy [147]. Thus, continued observation may be appropriate for most patients who have repeated attacks of uncomplicated diverticulitis.

**Systemic antibiotic treatment alone is usually the most appropriate treatment for patients with a small (<4 cm in diameter) diverticular abscess and image guided percutaneous drainage is for those with a large (>4 cm in diameter) one (Recommendation 2 B)**.

For patients in whom diverticulitis is complicated by peridiverticular abscess formation, the size of the abscess is an important determinant of the need for percutaneous drainage [145]. Many patients who have small pericolic abscesses (4 cm or less in diameter) without peritonitis (Hinchey stage 1) can be treated conservatively with bowel rest and broad-spectrum antibiotics [148]. For patients with peridiverticular abscesses that are larger than 4 cm in diameter (Hinchey stage 2), observational studies indicate that CT-guided percutaneous drainage can be beneficial [149-160]. This procedure typically eliminates or reduces the size of the abscess [148,151,152], with a reduction in pain, resolution of leukocytosis, and defervescence usually seen within several days [153]. Percutaneous drainage may allow for elective rather than emergency surgery, increasing the likelihood of a successful one-stage procedure. Patients whose abscess cavities contain gross feculent material tend to respond poorly, and early surgical intervention is usually required.

**Elective colon resection should typically be advised if an episode of complicated diverticulitis is treated non-operatively (Recommendation 2 C)**.

After percutaneous drainage of a diverticular abscess, a later colectomy usually should be planned, because 41 percent of patients will otherwise develop severe recurrent sepsis [154]. Some, but not all, retrospective studies suggest that the number of recurrences is associated with the chance that emergency surgery will be required at some point in the future [155].

**The resection should be carried proximally to the compliant bowel and extend distally to the upper rectum (Recommendation 1 C)**.

It is usually sufficient to remove only the most severely affected segment; however, the proximal margin of resection should be in an area of pliable colon without hypertrophy or inflammation [137]. Not all of the diverticula-bearing colon must be removed. Usually a sigmoid colectomy will suffice; however, occasionally the proximal resection margin must extend well into the descending colon or to the left transverse colon. Distally, the margin of resection should be where the taenia coli splay out onto the upper rectum. After sigmoid colectomy for diverticulitis, an important predictor of recurrent diverticulitis is a colosigmoid rather than a colorectal anastomosis [156,157].

**When a colectomy for diverticular disease is performed, a laparoscopic approach is appropriate in selected patients (Recommendation 1 B)**.

Laparoscopic colectomy may have advantages over open laparotomy, including less pain, smaller scar, and shorter recovery [137]. There is no increase in early or late complications [158,159]. Cost and outcome are comparable to open resection [160]. Laparoscopic surgery is acceptable in the elderly [161] and seems to be safe in selected patients with complicated disease [162].

**Urgent operation is required for patients with diffuse peritonitis or for those who fail non-operative management of acute diverticulitis (Recommendation 1 B)**.

If a patient presents with severe or diffuse peritonitis, emergency colon resection is necessary. Also, if sepsis does not improve with inpatient conservative treatment of acute diverticulitis or after percutaneous drainage, surgery is indicated [137]. Immunosuppressed or immunocompromised patients are more likely to present with perforation or fail medical management, so a lower threshold for urgent or elective surgery should apply to them [163]. The source control of diffuse peritonitis is discussed together in the next topic of large bowel perforations.

#### Large bowel perforations

No practice guideline has been proposed for the source control of large bowel perforation.

Causes of large bowel perforations include (1) penetrating foreign body perforation, (2) extrinsic bowel obstruction, (3) intrinsic bowel obstruction, (4) direct loss of bowel wall integrity without foreign body perforation, (5) intestinal ischemia, and (6) infection.

The principles of source control include: control of the site of perforation, evacuation of contamination, debridement of necrotic tissue, and re-establishment of functional anatomy. Many patients who have large bowel perforations develop sepsis with accompanying hemodynamic compromise, hypothermia, acidosis, and a coagulopathy [164]. These patients require rapid resuscitation and rapid surgery. The standard approach is known as damage control surgery. The goal is to rapidly obtain source control and peritoneal toilet without prolonging the surgery to restore functional anatomy or extensively mobilize tissues [165].

The decision whether to perform a proximal diverting procedure is based on the surgeon's assessment of the risks of anastomotic breakdown and other complications such as the patient's nutritional status, the quality of the tissues, the amount of bowel contamination, the extent of blood loss, and the intraoperative stability of the patient's condition [135,166].

**Hartmann's procedure may be performed for the treatment of large bowel perforations (Recommendation 2 C)**.

Two-stage procedures are typically used in emergency situations with fecal peritonitis and in most cases with purulent peritonitis. A common approach is the Hartmann's procedure, which involves resection of the diseased colon, an end-colostomy, and creation of a rectal stump; this is followed by colostomy closure several months later [167,168]. Reversal of Hartmann's procedure is also associated with substantial morbidity and even mortality [169]. It is well known that patients with stomas may face both physical and psychological difficulties [170,171].

**Primary anastomosis with or without proximal diverting stoma may be performed in selected patients (Recommendation 2 C)**.

It appears that resection and primary anastomosis, with or without proximal diverting stoma (colostomy or ileostomy), can be safely undertaken in selected patients who have phlegmons, abscess formation with localized peritonitis, diffuse purulent peritonitis, obstruction, or fistula formation [145,166,172,173]. Although data are not available from randomized trials, observational studies that include matched patients suggest similar overall mortality rates and lower risks of wound infection and postoperative abscess formation with a one-stage approach [168]. On-table colonic lavage may also be considered [174].

### Antimicrobial therapy for extra-biliary community-acquired IAIs

Once the diagnosis of intra-abdominal infection is suspected, it is necessary to begin empiric antimicrobial therapy.

However routine use of antimicrobial therapy is not appropriate for all patients with intra-abdominal infections.

In uncomplicated IAIs, when the focus of infection is treated effectively by surgical excision of the involved tissue, the administration of antibiotics is unnecessary beyond prophylaxis [175].

In complicated IAIs, when infectious process proceeds beyond the organ, causing either localized peritonitis (intra-abdominal abscess), or diffuse peritonitis antimicrobial therapy is mandatory.

**The choice of antimicrobial regimen depends on the source of intra-abdominal infection, risk factors for specific microorganisms and resistance patterns and clinical patient's condition (Recommendation 1 C)**.

The principles of empiric antibiotic treatment should be defined according to the most frequently isolated germs, always taking into consideration the local trend of antibiotic resistance.

The major pathogens involved in community-acquired IAIs are Enterobacteriaceae, streptococci and anaerobes. The main resistance problem is represented by ESBL producers Enterobacteriaceae, even today frequently found in community acquired infections.

Many factors can raise the risk of selection of ESBL but prior exposition to antibiotics (mainly third generation cephalosporins) and comorbidities that make frequent the exposure of patients to multiple antibiotic treatments, are the most significant [1,176,177].

Many others factors can contribute to the severity of an intra-abdominal infection and to a patient's risk for a poor outcome, like patient age, underlying co-morbidities, extent of infection, nutritional status and the success of initial source control procedures. Dividing patients with intra-abdominal infections into lower and higher risk categories is not always simple, but attempting to assess a patient's risk of treatment failure is essential to optimize a treatment plan. In this context adding a standardized evaluation of the clinical condition, represented by the sepsis grading, may be extremely helpful.

In fact in critically ill patients the possibility that the normal flora may be modified and that the IAI could be caused by several unexpected pathogens and by more resistant flora must be considered. In these patients antimicrobial regimens with broader spectrum of activity are recommended.

Therefore in a stable and low risk patient a simpler antibiotic choice, not including ESBL in the spectrum of activity is correct, while in critical and high risk patients any antibiotic regimen must take into account the risk of ESBL.

The available therapeutic options for the treatment of ESBL-associated infections are limited by drug resistance conferred by the ESBLs. The frequently observed co-resistances include various antibiotic classes (fluoroquinolones, aminoglycosides, tetracyclines, and trimethoprim/sulfamethoxazole). Carbapenems, stable against hydrolyzing activity of ESBLs, are considered as the drug of choice for the treatment of these infections. Tigecycline and polymyxins have a strong in vitro antimicrobial activity against ESBL-producing bacteria, and the first should be considered a reasonable alternative. This is particularly true from an epidemiological point of view; in fact today any large hospital should implement carbapenems-sparing stewardship programs to control the spread of carbapenemase producing gram negative bacteria.

Although in the prospective French survey by Montravers and coll, a higher percentage of isolation of *Enterococcus faecalis *in non surviving patients was reported (23% versus 9%) [35], empirical treatment against Enterococci and has not been generally recommended for patients with community-acquired IAI. In fact in several clinical trials comparing different therapeutic options inclusion/exclusion of agents with enterococcal coverage provides no impact in outcomes for patients with community-acquired infections [178,179].

In the setting of community acquired IAIs, antimicrobial therapy for enterococci should be considered on a patient-by-patient basis, mainly in high risk patients, in immunocompromised patients and in patients with valvular heart disease or prosthetic materials [180].

Methicillin resistant *Staphylococcus aureus *(MRSA) is not commonly isolated from patients with community-acquired intra-abdominal infection. Therefore empirical treatment against MRSA is not recommended in this setting.

Normally empiric antifungal therapy for *Candida *is not recommended for adult and pediatric patients with community acquired intra-abdominal infection with the exclusion of immunocompromised patients (because of neutropenia, and receipt of immunosuppressive agents, including glucocorticosteroids, chemotherapeutic agents, and immunomodulators) and in patients recently exposed to broad spectrum antimicrobials. However, considering the aforementioned high morality rate of candida peritonitis [38], considering an antifungal coverage in critically ill patients should be correct.

Community-acquired IAIs may be managed with either single or multiple antimicrobial regimens, in relation to the need to ensure a spectrum of antimicrobial activity more or less wide.

Beta-lactam/beta-lactamase inhibitor combinations have an in vitro activity against gram-positive, gram-negative and anaerobe organisms [181,182] and are still reliable option for the empiric treatment of IAIs [183]. However, the increasing resistance of *Enterobacteriaceae *reported in the last decade also among community-acquired infections restricts their empirical use to patients without risk factor for resistances [184].

In the past Cephalosporins have been often used in the treatment of intra-abdominal infections. Among third generation cephalosporins both subgroups with poor activity against *Pseudomonas aeruginosa *and with activity against *Pseudomonas aeruginosa *(cefepime and ceftazidime) have been used in the treatment of IAIs in association with metronidazole. Both cephalosporins have acquired resistance in enterobacteriaceae and intrinsic resistance in Enterococci [185-188]. In light of the emerging concern of ESBL producing enterobacteriaceae species due to selection pressure by increase use of cephalosporins, the routinely use of all cephalosporins should be discouraged.

Aztreonam is a parenteral synthetic beta-lactam antibiotic and the first monobactam to be marketed. Aztreonam exhibits potent and specific activity in vitro against a wide spectrum of Gram-negative aerobic pathogens including *Pseudomonas aeruginosa *but its use is burdened by the same problems of resistances to cephalosporins.

Carbapenems have a spectrum of antimicrobial activity that includes Gram-positives (except MDR resistant gram positive cocci) and Gram-negative aerobic and anaerobic pathogens. They are the preferred antimicrobial agents for ESBL and AmpC-producing organisms; however, their widespread use in outbreaks and endemic regions of these organisms has led to increased rates of carbapenem-resistant *P. aeruginosa *and *Acinetobacter sp*. In addition, the selection of intrinsically carbapenem-resistant organisms such as *Stenotrophomonas maltophilia *and vancomycin-resistant *Enterococcus faecium *can be seen [189].

Group 1 carbapenems includes ertapenem, a once a day carbapenem that shares the activity of imipenem and meropenem against most species, including extended-spectrum beta-lactamase (ESBL) - producing pathogens, but is not active against *Pseudomonas spp. *and *Enterococcus *[190,191].

Group 2 includes imipenem/cilastatin, meropenem and doripenem, that share activity against non-fermentative gram-negative bacilli. Slightly higher in-vitro activity against some strains of Pseudomonas sp. has been reported with doripenem in registrative trials [192].

Also fluoroquinolones have been widely used in the last years for the treatment of IAIs, because of their excellent activity against aerobic Gram-negative bacteria and tissue penetration. In addition all the fluoroquinolones are rapidly and almost completely absorbed from the gastrointestinal tract [193,194]. The combination of ciprofloxacin/metronidazole has been one of the most commonly used regimens for the treatment of patients with complicated IAIs in the last years. The last quinolone developed, Moxifloxacin, has shown activity against a wide range of aerobic Gram-positive and Gram-negative [195]. Compared with ciprofloxacin, moxifloxacin has enhanced activity against Gram-positive bacteria with a decrease in activity against Gram-negative bacteria [196]. Among quinolones moxifloxacin seems to be effective also against *Bacterioides fragilis*, suggesting that it may be effective without antianaerobic agents [197-199].

However, in recent years, the prevalence of resistance between Enterobacteriaceae and non-fermentative gram-negative bacilli has been so high as to make their use in empirical regimen not recommended.

Aminoglycosides are particularly active against aerobic Gram-negative bacteria and act synergistically against certain Gram-positive organisms. They are effective against *Pseudomonas aeruginosa *but not effective against anaerobic bacteria. The aminoglycosides may not be optimal for-the treatment of abscesses or intra-abdominal infections due to their low penetration in acidic environments [200]. Therefore they are not recommended for the routine empiric treatment of community-acquired IAIs and may be reserved for patients with allergies to b-lactam agents [1].

Tigecycline is a parenteral glycylcycline antibiotic derived from minocycline. It is the first representative of the glycylcycline class of antibacterial agents to be marketed for clinical use [201,202]. Tigecycline has no activity in vitro against *P. aeruginosa *and *P. mirabilis *but represents a significant treatment option for complicated IAIs due to its favorable in vitro activity against anaerobic organisms, Enterococci, several ESBL-producing *Enterobacteriaceae *and carbapenemase-producing *Enterobacteriaceae*, *Acinetobacter sp*. and *Stenotrophomonas maltophilia *[203-206].

The use of tigecycline in the abdominal infections is particularly attractive in view of its pharmacokinetics/pharmacodynamics properties. In fact the drug is eliminated by active biliary secretion, able to determinate very high biliary and fecal concentrations [207]. A study finalized to the determination of tissue and corresponding serum concentration of tigecycline at selected time points in several different body sites, performed in 104 subjects undergoing surgical or medical procedures, showed that concentration, expressed as the ratio of AUC0-24 was extremely high for bile [208].

Moreover a PD analysis based on the data of microbiological surveys, performed by the Montecarlo simulation, demonstrated a predicted cumulative response (PCR) fraction for Tigeciclyne in peritonitis over 95% for *E. coli *and *Enterococcus *and over 75% for *Klebsiella spp*, *Enterobacter spp *and *A. baumannii *[209]. Tigecycline (TGC) has demonstrated non-inferiority in terms of clinical efficacy and safety versus imipenem/cilastatin and combination regimen of Ceftriaxone/metronidazole in Phase 3 clinical trials for complicated intra-abdominal infection [210,211].

But the greater significance of the use of tigecycline in empirical antibiotic regimens for IAIs is related to the possibility of saving carbapenems prescriptions. From an epidemiological point of view tigecycline should be a qualified therapeutic option in a carbapenems-sparing stewardship programs, as extended-spectrum b-lactamases become widely disseminated among the endogenous gut *Enterobacteriaceae*.

Distinguishing antimicrobial regimens according to the clinical patient's severity, the presumed pathogens and risk factors for major resistance patterns, the presumed/identified source of infection it is possible to standardize the empirical approach to the main clinical condition related to IAIs.

In appendices 1, 2, 3, 4 are summarized the antimicrobial regimens for extrabiliary community-acquired IAIs, recommended by WSES consensus conference.

**Since the causative pathogens and the related resistance patterns can not easily be predicted (higher-risk patients), cultures from the site of infection must be always obtained (Recommendation 1 B)**.

Although the absence of impact of bacteriological cultures has been documented, especially in appendicitis, in this era of the broad spread of resistant microorganisms such as nosocomial and community extended-spectrum b-lactamase (ESBL) Enterobacteriaceae, carbapenemase producing gram negatives, b lactam- and vancomycin resistant enterococci (VRE), the threat of resistance is a source of major concern for clinicians. Therefore the results of the microbiological analyses have great importance for the therapeutic strategy of every patient, in particular in the adaptation of the initial antibiotic treatment, and at the same time are of paramount importance to ensure adequacy of empirical antimicrobial treatment.

The habit of considering the microbiological diagnosis useless is probably responsible for the relative scarcity of microbiological data and the variability of results of the few studies appropriate to emphasize the changes in resistance in IAI patients.

Cultures should be performed at least from intra-abdominal samples from surgery or interventional drainage procedures, providing sufficient volume (at least 1 mL of fluid or tissue, preferably more) and sending them to the laboratory using an appropriate transport system.

## Biliary Community-Acquired Intra-Abdominal infections

### Source control

Recent guidelines have been published for the management of acute cholecystitis and acute cholangitis [212-214].

#### Cholecystitis

**Laparoscopic cholecystectomy has been accepted as an effective and safe treatment for acute cholecystitis (Recommendation 1 A)**.

Laparoscopic cholecystectomy versus open cholecystectomy question has been extensively investigated. Beginning in the early 1990s, techniques and indications for laparoscopic management of the acutely inflamed gallbladder were discussed and laparoscopic cholecystectomy is now accepted as being safe for acute cholecystitis.

Many RCTs have demonstrated that laparoscopic cholecystectomy is effective and safe for acute cholecystitis [215-220].

In the Johansson and coll. randomized clinical trial there were no significant differences beetwen laparoscopic cholecystectomy and open cholecystectomy, in rate of postoperative complications, pain score at discharge and sick leave.

Seventy patients who met the criteria for acute cholecystitis were randomized to open or laparoscopic cholecystectomy. In eight patients a laparoscopic procedure was converted to open cholecystectomy. Median operating time was 90 (range 30-155) and 80 (range 50-170) min in the laparoscopic and open groups respectively (P = 0.040). The direct medical costs were equivalent in the two groups. Although median postoperative hospital stay was 2 days in each group, it was significantly shorter in the laparoscopic group (P = 0.011).

In the Kiviluoto and coll. randomized clinical trial there were no deaths or bile-duct lesions in either group, but the postoperative complication rate was significantly (p = 0.0048) higher in the open cholecystectomy than in the laparoscopic cholecystectomy group: seven (23%) patients had major and six (19%) minor complications after OC, whereas only one (3%) minor complication occurred after LC. The postoperative hospital stay was significantly shorter in the LC than the OC group (p = 0.0063).

**Early laparoscopic cholecystectomy during acute cholecystitis appears safe and shortens the total hospital stay when it is compared with delayed laparoscopic cholecystectomy (Recommendation 1 A)**.

The most important innovation in the surgical treatment of acute gallstone cholecystitis (AGC) concerns timing.

Several RCTs and meta-analyses [221-224] have showed that, compared with delayed laparoscopic cholecystectomy, early laparoscopic cholecystectomy for acute cholecystitis reduces the total length of hospital stay and the risk of readmissions attributable to recurrent acute cholecystitis. It is a more cost-effective approach for the management of acute cholecystitis.

In the Gurusamy and coll. meta-analysis [221] there was no significant difference between early and delayed groups in terms of bile duct injury or conversion to open cholecystectomy. The total hospital stay was shorter by 4 days for early laparoscopic cholecystectomy.

In Siddiqui and coll. meta-analysis [222] there was no significant difference in conversion rates and postoperative complications between early and delayed groups. Operation time was significantly reduced with delayed cholecystectomy. The total hospital stay was significantly reduced with early cholecystectomy.

In order to analyze whether delay from onset of symptoms was related to the conversion rate in patients with a acute cholecystitis, a retrospective case note review of patients undergoing emergency cholecystectomy in a single institution between January 2002 and December 2005 was published on 2007 [225]. Early intervention for acute cholecystitis (preferably within 2 days of onset of symptoms) was most likely to result in successful laparoscopic cholecystectomy; increasing delay was associated with conversion to open surgery.

**The use of percutaneous cholecystostomy in critically ill patients with acute cholecystitis is both safe and effective (Recommendation 2 B)**.

There are no randomized studies evaluating the outcome of percutaneous cholecystostomy vs. cholecystectomy. It is not possible to make definitive recommendations regarding treatment by PC or cholecystectomy in elderly or critically ill patients with acute cholecystitis. The use of percutaneous cholecystostomy in critically ill patients with acute cholecystitis is both safe and effective.

Whenever possible, percutaneous cholecystostomy should be followed by laparoscopic cholecystectomy.

A systematic electronic database search was performed on the subject of percutaneous cholecystostomy (PC) in the elderly population [226].

Successful intervention was seen in 85.6% of patients with acute cholecystitis. A total of 40% of patients treated with PC were later cholecystectomized, with a mortality rate of 1.96%. Procedure mortality was 0.36%, but 30-day mortality rates were 15.4% in patients treated with PC and 4.5% in those treated with acute cholecystectomy (P < 0.001).

**Early diagnosis of gallbladder perforation and immediate surgical intervention may decrease morbidity and mortality (Recommendation 1 C)**.

Gallbladder perforation is an unusual initial presentation of gallbladder disease. Early diagnosis of gallbladder perforation and immediate surgical intervention are of prime importance in decreasing morbidity and mortality associated with this condition.

It is rarely diagnosed preoperatively. Late operative intervention is associated with increased morbidity, mortality, number of ICU admissions, and long postoperative hospital stays [227-230].

#### Cholangitis

Biliary drainage is a radical method to relieve cholestasis, a cause of acute cholangitis, and takes a central part in the treatment of acute cholangitis.

Biliary drainage can be achieved by three different procedures:

• Endoscopic

• Percutaneous transhepatic

• Open drainage

It has been reported that when no appropriate biliary drainage was available 20-30 years ago, the mortality of acute cholangitis with conservative treatment was extremely high. There has been no randomized controlled trial (RCT) comparing conservative treatment and biliary drainage. However, many patients with acute cholangitis cannot be treated by conservative treatment alone [231,232].

**Endoscopic drainage is safer and more effective than open drainage. (Recommendation 1 A)**.

A randomized controlled trial (RCT) was conducted to compare endoscopic and open drainage in 82 patients with severe acute cholangitis with hypotension and disturbed consciousness. This RCT demonstrated that the morbidity and mortality of endoscopic naso-biliary drainage (ENBD) + endoscopic sphincterotomy (EST; *n *= 41) were significantly lower than those of T-tube drainage under laparotomy (*n *= 41). The Authors concluded that morbidity and mortality of endoscopic naso-biliary drainage (ENBD) + endoscopic sphincterotomy are lower than those of T-tube drainage under laparotomy [233].

**Endoscopic modalities currently are favored over percutaneous procedures because of a lower risk of complication. There is no RCT comparing endoscopic and percutaneous drainage (Recommendation 2 C)**.

Considering the rare occurrence of serious complications such as intraperitoneal hemorrhage and biliary peritonitis, and the shorter duration of hospitalization, endoscopic drainage is preferred whenever it is available and applicable [234-237].

**Open drainage should only be used in patients for whom endoscopic or percutaneous transhepatic drainage is contraindicated or those in whom it has been unsuccessfully performed. (Recommendation 2 C)**.

There is no RCT comparing open drainage and endoscopic or percutaneous drainage [238].

### Antimicrobial therapy for biliary infections

Antibiotics are always recommended in complicated cholecystitis and in delayed treatment of uncomplicated cholecystitis.

In uncomplicated cholecystitis, when the focus of infection is treated effectively by cholecystectomy, the administration of antibiotics is unnecessary beyond prophylaxis.

Patients with an infected focus that can be eradicated effectively by surgical intervention can potentially be treated with only 24 hours of antimicrobial prophylaxis.

**The most important factors for antimicrobial drug selection in biliary infections are antimicrobial activity against causative bacteria, clinical patient's condition and biliary levels of the antimicrobial agents (Recommendation 1 B)**.

**Knowledge of the secretion of antibiotics into bile and of their efficacy should be helpful in choosing the best therapeutic regimen (Recommendation 1 C)**.

Organisms most often isolated in biliary infections are the gram-negative aerobes, *Escherichia coli *and *Klebsiella pneumonia *and anaerobes, especially *Bacteroides fragilis*. Activity against enterococci is not required since their pathogenicity in biliary tract infections remains unclear [239-241].

The efficacy of antibiotics in the treatment of biliary infections depends on effective biliary antibiotic concentrations [242-245].

It has been debated whether antimicrobials with good biliary penetration should be recommended for biliary infections. However, there are no clinical or experimental data to strongly support the recommendation of antimicrobials with excellent biliary penetration for these patients. Other important factors include the antimicrobial potency of individual compounds, and the effect of bile on antibacterial activity [246].

Penicillins are still frequently used in biliary infections. Aminopenicillins such as amoxicillin are excreted unchanged in the bile. In patients with normal function of biliary tract, amoxicillin bile concentrations are higher than the serum concentrations (3 rates higher than the concentrations in plasma).

Fluoroquinolones have excellent bioavailability; they are excreted by renal, hepatic and biliary excretion. Ciprofloxacin biliary concentrations are generally higher then the concentrations in the plasma (28 to 45 rates higher than the concentrations in plasma). Besides, ciprofloxacin has been proven to reach high biliary concentrations also in patients with obstruction due to the anticipated secretion of quinolone by biliary epithelium. An alternative to amoxicillin/clavulanate, ciprofloxacin plus metronidazole may be indicated for biliary infections, in no critically ill patient and in absence of risk factors for resistance patterns.

Piperacillin is the penicillin with highest rate of bile excretion (25% in active form). Bile concentrations are up to 60 rates higher than the concentrations in plasma. The combination of piperacillin with tazobactam further extends its spectrum. However tazobactam pharmacokinetics is different from piperacillin pharmacokinetics and during a regular therapy regimen employing piperacillin/tazobactam combination, tazobactam reaches effective concentrations in the bile only during the first 3 hours following its administration.

Glicilcyclines such as tigecycline have a broad spectrum of activity and a very good availability in the bladder wall and bile. Tigecycline is a very good antimicrobial option in biliary infections.

Also for biliary intra-abdominal infections WSES consensus conference distinguished antimicrobial regimens according to the clinical patient's condition and the risk factors for resistance patterns.

In appendices 5, 6, 7, 8 are summarized the antimicrobial regimens for biliary community-acquired intra-abdominal infections, recommended by WSES consensus conference.

## Hospital-acquired intra-abdominal infections

Hospital-acquired intra-abdominal infections are infections not present on admission that become evident 48 hours or more after admission in patients hospitalized for a reason other than intra-abdominal infection [247].

Both post-operative and non post-operative nosocomial intra-abdominal infections are associated with increased mortality due to underlying patient health status and increased likelihood of infection caused by MDR organisms [248-255].

The main clinical differences between the patients with community-acquired intra-abdominal infections and patients with nosocomial intra-abdominal infections are [35]:

• higher proportion of underlying disease

• severity criteria at the time of diagnosis for nosocomial cases

The most common cause of postoperative peritonitis is anastomotic failure/leak.

In few instances of postoperative peritonitis, the anastomosis may be intact; however, the patient may remain sick because of residual peritonitis. Among them is the inadequate drainage of the initial septic focus, in which the surgeon failed to drain completely, or more commonly, the peritoneum does not have the sufficient defense capacity to control the problem.

Hospital acquired, non-postoperative IAIs, which arise in patients hospitalized for reasons unrelated to abdominal pathology, portend a particularly poor prognosis.

Diagnosis is often delayed due to both a low index of suspicion, poor underlying health status, and altered sensorium.

Non-postoperative nosocomial intra-abdominal infections are frequently characterized as severe infections diagnosed lately in fragile patients [254].

Prospective analysis of patients operated for secondary non-postoperative nosocomial intra-abdominal infections collected in 176 French study centers was published 2004 [254].

When compared with CAI patients, Non-PostopNAI patients presented:

• increased interval between admission to the surgical ward and operation

• increased proportions of underlying diseases

In non-PostopNAI patients, increased proportions of therapeutic failure and of fatalities were observed [254].

Unlike previous studies, recent studies observed no difference in incidence of prognosis between community-acquired and nosocomial intra-abdominal infections.

Riché and coll. [45] have prospectively studied a cohort of 180 consecutive patients operated on for generalized peritonitis.

There were 24 deaths among the 112 patients with community-acquired peritonitis (21% mortality rate) and 11 deaths among the 68 patients with postoperative peritonitis (16% mortality rate). Survival rates at day 30 were not statistically different for community-acquired and postoperative peritonitis. The proportion of patients operated less than 24 hours after the onset of symptoms was not different between community-acquired and postoperative peritonitis (54% *vs*. 49%, respectively; *P *= 0.61).

In the Inui and coll. [256] study, 452 patients, 234 (51.8%) had CIAIs and 218 (48.2%) had NIAIs, treated at a single urban academic hospital over 8 years (June 1999-June 2007) were retrospectively reviewed.

When patients with appendicitis were excluded, there was no difference in mortality or complications between patients with CIAIs and NIAIs.

Source control represents a key component of success in therapy of sepsis. It includes drainage of infected fluids, debridement of infected soft tissues, removal of infected devices or foreign bodies, and finally, definite measures to correct anatomic derangement resulting in ongoing microbial contamination and to restore optimal function.

Recommendations have low grade due to the difficulty to perform appropriate randomized clinical trials in this respect.

**Percutaneous abscess drainage should be the primary procedure to treat postoperative localized intra-abdominal abscess without signs of generalized peritonitis (Recommendation 2 C)**.

Some retrospective studies in the surgery and radiology literature have documented the effectiveness of percutaneous drainage to treat postoperative localized intra-abdominal abscess [257-259].

**Source control should be obtained as early as possible after the diagnosis of postoperative intra-abdominal peritonitis has been confirmed. Inability to control the septic source is associated significantly with increase in mortality (Recommendation 1 C)**.

Inability to control the septic source is associated significantly with increase in mortality.

Delaying relaparotomy for more than 24 h or the presence of organ failure result in higher mortality in postoperative intra-abdominal infections.

The value of physical tests and laboratory parameters in diagnosing abdominal sepsis is limited. CT-scanning revealed the highest diagnostic accuracy. Early relaparotomy appears to be the most reasonable option to treat postoperative peritonitis [260].

### Re-laparotomy strategy

Some patients are prone to persisting intra-abdominal infection regardless of eradication of the source of infection and timely relaparotomy provides the only surgical option that significantly improves outcome.

In these cases single operation may not be sufficient to achieve source control, thus re-exploration is necessary [261-263].

The decision to and when to perform a relaparotomy in secondary peritonitis is largely subjective and based on professional experience. Factors indicative of progressive or persistent organ failure during early postoperative follow-up are the best indicators for ongoing infection and associated positive findings at relaparotomy [264-266].

Three methods of local mechanical management of abdominal sepsis following initial laparotomy for source control are currently debated:

(1) Open-abdomen

(2) planned relaparotomy

(3) on-demand relaparotomy

On demand relaparotomy may be considered the preferred surgical strategy in patients with severe peritonitis because it has a substantial reduction in relaparotomies, health care utilization, and medical costs. (Recommendation 1 A)

In 2007 van Ruler and coll. [267] published a randomized, clinical trial comparing on-Demand Vs Planned Relaparotomy strategy in patients with severe peritonitis.

In the van Ruler trial a total of 232 patients with severe intra-abdominal infections (116 on-demand and 116 planned) were randomized.

In planned relaparotomy group, relaparotomies were performed every 36 to 48 hours after the index laparotomy to inspect, drain, lavage, and perform other necessary abdominal interventions for residual peritonitis or new infectious focus.

In on-Demand relaparotomy group, relaparotomies were only performed in patients with clinical deterioration or lack of clinical improvement with a likely intra-abdominal cause.

Patients in the on-demand relaparotomy group did not have a significantly lower rate of adverse outcomes compared with patients in the planned relaparotomy group but did have a substantial reduction in relaparotomies, health care utilization, and medical costs.

Patients in the on-demand group had shorter median intensive care unit stays (7 vs 11 days; *P *= .001) and shorter median hospital stays (27 vs 35 days; *P *= .008). Direct medical costs per patient were reduced by 23% using the on-demand strategy.

Some studies have investigated open abdomen in intra-abdominal infections and generated great interest and hope [268-270].

In 2007 a randomized study by Robledo and coll. [271] compared open with closed "on demand" management of severe peritonitis.

During a 24-month period, 40 patients with SSP were admitted for treatment. Although the difference in the mortality rate (55% vs. 30%) did not reach statistical significance (p < 0.05; chi-square and Fisher exact test), the relative risk and odds ratio for death were 1.83 and 2.85 times higher in open abdomen patients group. This clinical finding, as evidenced by the clear tendency toward a more favorable outcome for patients in closed open group, led to termination of the study at the first interim analysis.

This randomized study from a single institution demonstrates that closed management of the abdomen may be a more rational approach after operative treatment of SSP and questions the recent enthusiasm for the open alternative, which has been based on observational studies. However in this study, the "open abdomen" was managed with a non-absorbable polypropylene mesh, without topical negative pressure.

### Antimicrobial treatment of hospital-acquired intra-abdominal infections

Hospital-acquired IAIs are among the most difficult infections to diagnose early and treat effectively.

A successful outcome depends on early diagnosis, rapid and appropriate surgical intervention, and the selection of effective antimicrobial regimens.

**Hospital acquired infections are commonly caused by larger and more resistant flora, and for these infections, complex multidrug regimens are always recommended (Recommendation 1 B)**.

The threat of antimicrobial resistance has been identified as one of the major challenges in the management of complicated IAIs and was already discussed in the previous chapter.

In order to describe the differences in microbiological and resistance patterns between community-acquired and nosocomial intra-abdominal infections a prospective, observational multicentric study (EBIIA) was completed in French [35]. From January to July 2005, patients undergoing surgery/interventional drainage for IAIs with a positive microbiological culture were included by 25 French centers. A total of 829 microorganisms were cultured.

In this study the number of peritoneal microorganisms per sample was ≥3 in 34% and 54% of cases, respectively, for community-acquired and nosocomial infections (P < 0.001). The distribution of the microorganisms differed according to the nosocomial or community origin of the infection but not according to their location (data not shown). In nosocomial patients, increased proportions of *Enterococcus faecalis *(33% versus 19% in community-acquired patients; P < 0.05) and Pseudomonas aeruginosa strains (13% versus 5% in community-acquired patients; P < 0.01) were observed. Conversely, in nosocomial patients, decreased proportions of *Escherichia coli *(52% versus 72% in community-acquired patients, P < 0.001) and streptococci strains were reported (31% versus 50% in community-acquired patients, P < 0.01).

Therefore the inclusion of anti-enterococcal drugs in any empirical antibiotic regimens in severe nosocomial IAIs and/or in patients with well known risk factors, seems appropriate, mainly if directed against *E. faecalis*.

Empiric therapy directed against vancomycin-resistant *Enterococcus faecium *is not recommended unless the patient is at very high risk for an infection due to this organism, such as a liver transplant recipient with an intra-abdominal infection originating in the hepatobiliary tree or a patient known to be colonized with vancomycin-resistant *E. faecium*.

*Enterococcus *infections are difficult to treat because of both intrinsic and acquired resistance to many antibiotics.

Enterococci are intrinsically resistant to many penicillins, and all cephalosporins with the possible exception of ceftobiprole and ceftaroline, currently undergoing clinical evaluation. Besides Enterococci have acquired resistance to many other classes of antibiotics, to which the organisms are not intrinsically resistant, including fluoroquinolones, aminoglycosides, and penicillins. Many strains of *E. faecalis *are susceptible to certain penicillins and glycopeptides; however, some strains of *E. faecium *may be resistant to these agents [272].

Vancomycin-resistant *Enterococcus *(VRE) infections have been associated with increased morbidity and mortality [273,274].

Many factors can increase the risk of colonization with VRE. These include previous antibiotic therapy (the number and duration of antibiotics received) prolonged hospitalization, hospitalization in an intensive care unit severity of illness, invasive procedures and devices, gastrointestinal surgery, transplantation, proximity to another VRE-positive patient [275].

Affected patients usually have multiple and relevant co-morbidities, with prolonged hospital stay and received long courses of broad spectrum antibiotics.

In the survey of Montravers and coworkers no differences in frequency of isolation of Candida spp were identified in community or hospital acquired IAIs, and the overall prevalence was under 5%, in contrast with other observations, especially those related to patients with recurrent gastrointestinal perforation/anastomotic leakage [276,277].

Although the epidemiological role of Candida spp in nosocomial peritonitis is not yet defined, the clinical role is significant, because Candidal isolation is normally associated to a poor prognosis.

The same study group on 2006 published an elegant retrospective, case-control study conducted in critically ill patients admitted to 17 French ICUs where the yielding of Candida spp from peritoneal specimen was a variable independently associated to mortality in the setting of nosocomial peritonitis [37].

More recently Montravers and coll. reported a mortality rate of 38% in a prospective cohort of 93 patients admitted to ICU with candidal peritonitis [38].

Therefore, like for Enterococci, the inclusion of an anticandidal drug in the empiric regimen of severe nosocomial acquired IAIs, seems appropriate as confirmed by IDSA guidelines [1].

The recently published IDSA guidelines for the treatment of invasive candidiasis [278] don't comprise a chapter specifically dedicated to candidal peritonitis. However the expert panels generically favor the use of echinocandins as first line empirical therapy in severely ill patients, recommending fluconazole for less severe conditions.

Therefore, transferring this concept to the context of IAIs we might advise the proscription of echinocandins as first line treatment in severe nosocomial IAIs. The IDSA guidelines also recommend the transition from an echinocandin to fluconazole for patients clinically stable and who have isolates of *Candida spp *susceptible to fluconazole; so the final recommendation would be to start with an echinocandin and to de-escalate to fluconazole as soon as possible on a clinical or microbiological basis.

In appendices 9,10 are summarized the antimicrobial regimens for hospital-acquired intra-abdominal infections, recommended by WSES consensus conference.

## Conclusions

The timing and adequacy of source control is the most important issue in the management of intra-abdominal sepsis, because an inadequate and late operation may have a negative effect on the outcome.

Concomitant adequate empiric antimicrobial therapy further influences patients' morbidity and mortality. Inappropriate antibiotic therapy of intra-abdominal infections may result in poor patient outcome and the selection of an appropriate agent is a real challenge because of the emerging resistance of target organisms to commonly prescribed antibiotics.

It is demonstrated that a strategy of early goal-directed therapy decreases the in-hospital mortality of patients who are taken to the emergency department in septic shock. An organized approach to the haemodynamic support to sepsis includes use of fluid resuscitation, vasopressor therapy and inotropic therapy.

A multidisciplinary approach to the management of critically ill patients may be an important factor in the quality of care.

## Competing interests

The authors declare that they have no competing interests.

## Authors' contributions

MS, PV designed the study. MS, CT partecipated in collection and assembly of data. MS, PV, KK, GG wrote the manuscript. All authors read and approved the final manuscript.

## Appendices

### Appendix 1. Antimicrobial therapy for community-acquired extrabiliary IAI in no critically ill patient, in absence of risk factors for ESBL

Community-acquired extrabiliary IAI

No critically ill patient

No risk factors for ESBL

AMOXICILLIN/CLAVULANATE

Daily schedula: 2.2 g every 6 hours (Infusion time 2 hours)

OR (Allergy to beta-lactams):

CIPROFLOXACIN

Daily schedula: 400 mg every 8 hours (Infusion time 30 min)

+

METRONIDAZOLE

Daily schedula: 500 mg every 6 hours (Infusion time 1 hour)

### Appendix 2. Antimicrobial therapy for community-acquired extrabiliary IAI in no critically ill patient, in presence of risk factors for ESBL

Community-acquired extrabiliary IAI

No critically ill patient

Risk factors for ESBL

ERTAPENEM

Daily schedula: 1 g every 24 hours (Infusion time 2 hours)

OR

TIGECYCLINE

Daily schedula: 100 mg LD then 50 mg every 24 hours

(Infusion time 2 hours)

### Appendix 3. Antimicrobial therapy for community-acquired extrabiliary IAI in critically ill patient, in absence of risk factors for ESBL

Community-acquired extrabiliary IAI

Critically ill patient (± SEVERE SEPSIS)

No risk factors for ESBL

PIPERACILLIN/TAZOBACTAM

Daily schedula: 8/2 g LD then 16/2 g/die by continuous infusion or

4.5 g every 6 hours (infusion time 4 hours)

### Appendix 4. Antimicrobial therapy for community-acquired extrabiliary IAI in critically ill patient, in presence of risk factors for ESBL

Community-acquired IAI

Critically ill patient (± SEVERE SEPSIS)

Risk factors for ESBL

MEROPENEM

Daily schedula: 500 mg every 6 hours (Infusion time 6 hours)

OR

IMIPENEM

Daily schedula: 500 mg every 4 hours (Infusion time 3 hours)

+/-

FLUCONAZOLE

Daily schedula: 600 mg LD then 400 mg every 24 hours (Infusion time 2 hours)

### Appendix 5. Antimicrobial therapy for biliary IAI in no critically ill patient, in absence of risk factors for ESBL

Community-acquired biliary IAI

No critically ill patient

No risk factors for ESBL

AMOXICILLIN/CLAVULANATE

Daily schedula: 2.2 g every 6 hours (Infusion time 2 hours)

OR (Allergy to beta-lactams)

CIPROFLOXACIN

Daily schedula: 400 mg every 8 hours (Infusion time 30 min)

+

METRONIDAZOLE

Daily schedula: 500 mg every 6 hours (Infusion time 1 hour)

### Appendix 6. Antimicrobial therapy for biliary IAI in no critically ill patient, in presence of risk factors for ESBL

Community-acquired biliary IAI

No critically ill patient

Risk factors for ESBL

TIGECYCLINE

Daily schedula: 100 mg LD then 50 mg every 12 hours (Infusion time 2 hours)

### Appendix 7. Antimicrobial therapy for biliary IAI in critically ill patient, in absence of risk factors for ESBL

Community-acquired biliary IAI

Critically ill patient (± SEVERE SEPSIS)

No risk factors for ESBL

PIPERACILLIN/TAZOBACTAM

Daily schedula: 8/2 g LD then 16/2 g/die by continuous infusion or

4.5 g every 6 hours (infusion time 4 hours)

### Appendix 8. Antimicrobial therapy for biliary IAI in critically ill patient, in presence of risk factors for ESBL

Community-acquired biliary IAI

Critically ill patient (SEVERE SEPSIS)

Risk factors for ESBL

PIPERACILLIN

Daily schedula: 8 g by LD then 16 g by continuous infusion or

4 g every 6 hours (Infusion time 4 hours)

+

TIGECYCLINE

Daily schedula: 100 mg LD then 50 mg every 12 hours (Infusion time 2 hours)

+/-

FLUCONAZOLE

Daily schedula: 600 mg LD then 400 mg every 24 hours (Infusion time 2 hours)

### Appendix 9. Antimicrobial therapy for hospital-acquired IAI in no critically ill patient

Hospital acquired IAI

No critically ill patient (< SEVERE SEPSIS)

Risk factors for MDR pathogens

PIPERACILLIN

Daily schedula: 8 g by LD then 16 g by continuous infusion or

4 g every 6 hours (Infusion time 4 hours)

+

TIGECYCLINE

Daily schedula: 100 mg LD then 50 mg every 12 h (Infusion Time: 2 hours)

+

FLUCONAZOLE

Daily Schedula: 600 mg LD then 400 mg every 24 h (Infusion time: 2 hours)

### Appendix 10. Antimicrobial therapy for hospital-acquired IAI in critically ill patient

Hospital-acquired extrabiliary IAI

Critically ill patient (±SEVERE SEPSIS)

Risk factors for MDR pathogens

PIPERACILLIN

Daily schedula: 8 g by LD then 16 g by continuous infusion or

4 g every 6 hours (Infusion time 4 hours)

+

TIGECYCLINE

Daily schedula: 100 mg LD then 50 mg every 12 h (Infusion Time: 2 hours)

+

ECHINOCANDIN

caspofungin (loading dose of 70 mg, then 50 mg daily),

anidulafungin (loading dose of 200 mg, then 100 mg daily),

micafungin (100 mg daily)

OR

MEROPENEM

Daily Schedula: 500 mg every 6 h (Infusion time: 6 hours)

IMIPENEM

Daily Schedula: 500 mg every 4 h (Infusion time: 3 hours)

DORIPENEM

Daily Schedula: 500 mg every 8 h (Infusion time: 4 hours)

+

TEICOPLANIN

Daily Schedula: LD 12 mg/kg/12 h for 3 doses then 6 mg/kg every 12 h (with TDM corrections - PD target 20-30 mg/L)

Daily schedula: 16 g by continuous infusion or

4 g every 6 hours (infusion time 4 hours)

+

ECHINOCANDIN

caspofungin (loading dose of 70 mg, then 50 mg daily),

anidulafungin (loading dose of 200 mg, then 100 mg daily),

micafungin (100 mg daily)
